# Foundational Elements of Applied Simulation Theory: Development and Implementation of a Longitudinal Simulation Educator Curriculum

**DOI:** 10.7759/cureus.1002

**Published:** 2017-01-27

**Authors:** Michelle Chiu, Glenn Posner, Susan Humphrey-Murto

**Affiliations:** 1 Department of Anesthesiology, University of Ottawa; 2 Department of Innovation in Medical Education, University of Ottawa; 3 Department of Pain Medicine, University of Ottawa; 4 Department of Medicine, University of Ottawa

**Keywords:** simulation based medical education, faculty development, postgraduate medical education, curriculum development, simulation specialist training, simulation educator training, simulation, curriculum, curriculum development research

## Abstract

Simulation-based education has gained popularity, yet many faculty members feel inadequately prepared to teach using this technique. Fellowship training in medical education exists, but there is little information regarding simulation or formal educational programs therein. In our institution, simulation fellowships were offered by individual clinical departments. We recognized the need for a formal curriculum in educational theory. Kern’s approach to curriculum development was used to develop, implement, and evaluate the Foundational Elements of Applied Simulation Theory (FEAST) curriculum.

Needs assessments resulted in a 26-topic curriculum; each biweekly session built upon the previous. Components essential to success included setting goals and objectives for each interactive session and having dedicated faculty, collaborative leadership and administrative support for the curriculum. Evaluation data was collated and analyzed annually via anonymous feedback surveys, focus groups, and retrospective pre-post self-assessment questionnaires.

Data collected from 32 fellows over five years of implementation showed that the curriculum improved knowledge, challenged thinking, and was excellent preparation for a career in simulation-based medical education. Themes arising from focus groups demonstrated that participants valued faculty expertise and the structure, practicality, and content of the curriculum.

We present a longitudinal simulation educator curriculum that adheres to a well-described framework of curriculum development. Program evaluation shows that FEAST has increased participant knowledge in key areas relevant to simulation-based education and that the curriculum has been successful in meeting the needs of novice simulation educators. Insights and practice points are offered for educators wishing to implement a similar curriculum in their institution.

## Introduction

Simulation-based education is increasingly used in medical education, yet physicians have very little formal training in delivering these programs [1]. Faculty members have expressed feelings of inadequacy in their ability to teach in a simulated setting and their perceived inadequacy has had a detrimental effect on student learning [2]. Several intensive simulation instructor courses have been developed to address the needs of the faculty; participation in these courses increases the perceived ability of the instructor to use simulation-based techniques to teach trainees [3]. However, the brief nature of these stand-alone courses implies that topics cannot be re-visited for further discussion. A longitudinal approach, with multiple sessions held over time versus stand-alone courses, may allow opportunities for a deepened understanding and application of the principles and techniques learned during the curriculum [4].

Many institutions have begun to offer medical education fellowship training, but there is limited information regarding simulation-specific fellowship training [5-6]. Steinert, et al. reviewed 53 papers describing postgraduate training in medical education, ranging from short workshops to longitudinal fellowship training [7]. Only 10% of the programs reviewed were longitudinal fellowship programs, none of which were focused on simulation-based medical education. Kotal, et al. identified 17 simulation-based fellowship programs in the USA; the program characteristics were described, but no details were provided on the existence of formal educational curricula [8]. Kinnear, et al. described a consensus-based approach to the proposed content and design of a simulation instructor curriculum, but they do not describe implementation of the course or program evaluation [9].

Kern’s six-step approach to curriculum development is widely used in medical education and is outlined in Table 1 [10]. We describe, using Kern’s framework, the development, implementation, and evaluation of the Foundational Elements of Applied Simulation Theory (FEAST) curriculum. Informed consent was obtained from all participants in this study.

**Table 1:** Kern’s Six Steps to the Development of Educational Curricula (10)

Kern’s Six Steps to Curriculum Development
Step 1: Problem Identification and General Needs Assessment
Step 2: Learner Targeted Needs Assessment
Step 3: Goals and Objectives Broad goals Specific objectives
Step 4: Educational Strategies Curriculum content Educational methods
Step 5: Implementation Identify resources (personnel, time, facilities) Obtain stakeholder support Develop administrative mechanisms (structure, communication, operations) Address barriers
Step 6: Evaluation and Feedback Individual learner Overall program

## Technical report

### Problem identification and needs assessment

Development of the FEAST curriculum began as a series of informal discussions within the Department of Anesthesiology between the simulation fellowship director, simulation faculty, and their simulation fellow. It was felt that beyond hands-on experience, deliberate instruction on various topics in simulation-based education would enhance the experience of the fellow. As the Departments of Emergency Medicine, Obstetrics/Gynecology, Surgery and Anesthesiology began collaborating more actively, it became evident that several departments were launching simulation fellowship programs. All departments identified a need for formal training in educational theory in addition to practical training and were interested in collaborating to generate and deliver a longitudinal curriculum.

To determine curriculum content, needs assessments were conducted amongst current and past fellows, 12 multi-disciplinary simulation educators (representing anesthesiology, surgery, emergency medicine, obstetrics/gynecology, internal medicine, and critical care) and the senior leadership of the University of Ottawa Skills and Simulation Center (uOSSC). Topics taught in existing simulation instructor courses were also reviewed. We identified 21 topics for the first iteration of FEAST. We performed annual program evaluations, which resulted in additional topics being added. Table 2 shows the current iteration of the FEAST curriculum, which consists of 26 topics in simulation-based education.

**Table 2:** FEAST Curriculum Themes & Session Topics* * This table does not depict the order in which sessions were delivered in the curriculum.

FEAST Curriculum Themes & Session Topics
Orientation
Welcome to your Fellowship in Simulation & Medical Education
Introduction to Audio Visual & Patient Simulation Equipment
Introduction to Simulation Centre Administration & Operations
Educational Principles & Learning Theory
History of Simulation in Medical Education
Educational Theory and Simulation
Curriculum Design in Simulation
Designing Needs Assessments
Conducting Programmatic Evaluation
High Stakes Assessment in Simulation
Simulation Instructor Training
Principles of Crisis Resource Management
Simulation Scenario Development
Debriefing I: How to Survive Your First Debrief
Debriefing II: Theory in Practice
The Difficult Debrief
Debriefing the Debriefer
Half-Marathon Debrief the Debriefer
Marathon Debrief the Debriefer
Simulation-based Team Training
Interprofessional Simulation
Interdisciplinary Simulation
Conflict Resolution Techniques
Human Factors & Human Error
Technical Skills Simulation
Introduction to Surgical Skills Models, Props & Moulage
Introduction to Technical Skills Simulation
Research Skills
Research in Medical Education I
Research in Medical Education II
Research in Medical Education III

### Goals and objectives

The goal of the FEAST curriculum is to provide simulation fellows with a solid foundation in the theory and practice of simulation via interactive teaching about instructional design, curriculum development, and research as it pertains to simulation-based medical education. Each interactive teaching session included topic-specific objectives to be attained (see Appendix).

### Educational strategies

Senior uOSSC staff and expert simulation educators from the Departments of Anesthesiology, Critical Care, Emergency Medicine, Obstetrics/Gynecology, and Surgery selected topics in which they had self-identified expertise. Sessions were given bimonthly from September to June of each year. Each interactive 90-minute session was followed by a facilitated 60-minute “Debrief the Debriefer” video review, where fellows could review videos of their own scenario debriefing with their colleagues and a faculty member. Fellows accumulated practical experience as an instructor during weekly simulation teaching sessions within their own specialty. In addition, four months into their fellowship, fellows facilitated simulation sessions outside their specialty area (for example, an anesthesiology simulation fellow would lead the obstetrics/gynecology simulation session). This innovative “fellow swap” allowed fellows to practice their debriefing skills in other content areas.

### Curriculum implementation

Personnel and time were essential resources for the success of this curriculum. A designated director and an administrative coordinator were vital. The director was responsible for overseeing implementation and scheduling of the curriculum, while the coordinator was responsible for managing the schedule, evaluation forms, and communication with faculty and fellows. Other key personnel included faculty with simulation expertise and a desire to teach. The faculty needed time to prepare and present their talks, and the fellows needed protected time to attend talks and complete pre-assigned readings. With up to 18 faculty members and 4–10 fellows per year, an effective administrative and communication structure amongst stakeholders was crucial.

FEAST lectures were given at the simulation center in conference rooms with access to computers, internet, and projectors. For some lectures, access to the surgical skills laboratory, simulation mannequins, and audiovisual equipment were necessary.

At the time of our needs assessment, we recognized the continuing need for administrative and political support. At our university, the Faculty of Medicine’s Medical Education Research/Innovation Unit is called the Department of Innovation in Medical Education (DIME). Their mandate is to promote educational innovation and scholarship. Partnering with DIME, which had an established fellowship in medical education and mutual interest in providing all fellows with training in learning theory, allowed for the allocation of further resources for administrative support. FEAST was incorporated as a mandatory component of the new DIME/uOSSC Fellowship in Medical Education/Simulation.

Additional support was given by the uOSSC, where senior management participated as faculty and provided access to physical space for training. Simulation educators supported the curriculum by providing their time and expertise in creating and delivering interactive presentations. The faculty recognized that their contributions to FEAST were mutually beneficial, as their time investment would be matched by contributions from colleagues towards the education of their fellows. All faculty received formal evaluations to be included in their teaching dossiers.

Several challenges were encountered during the implementation of this curriculum. Barriers and their solutions are shown in Table 3.

**Table 3:** Barriers Encountered and their Solutions during FEAST Implementation

Barrier	Solution
Suitable lecture timing	Varying clinical schedules amongst different specialties made it challenging to find a suitable time. Resolution of this issue was achieved via fellowship committee meetings and acknowledgement that FEAST was an essential fellowship component.
Absenteeism	Taking attendance, prompt notification and involvement of the FEAST and fellowship directors was effective at remediating this issue.
Inconsistent “Debriefing the Debriefer” sessions	Fellows reported that the weekly scheduled sessions did not occur regularly. Fellows taking a more active role, and reminders sent to faculty were effective strategies.
Research project initiation	Feedback from the fellows reflected their desire to have the research lectures earlier in the year. In subsequent iterations, these lectures were held over the summer months so that projects could be initiated earlier.
Increasing demand on enrollment	In recent years, several requests have been received for ad hoc participation in the program. These requests were denied as the curriculum was designed to build upon preceding lectures and the interactive nature of the presentations required a cohesive small group setting.

### Curriculum evaluation

The FEAST Curriculum was introduced in 2011. We collected evaluation data from 32 fellows over the first five years via anonymous feedback surveys, focus groups, and retrospective pre-post self-assessment questionnaires. Institutional research ethics board review was waived as this qualified as program evaluation. A 5-point Likert scale was used and the mean scores are reported for selected categories.

Feedback Surveys

The fellows indicated that the FEAST curriculum was given at an “appropriate depth and level” (4.5 out of 5; 1=strongly disagree, 5=strongly agree), the “instructional format of the FEAST curriculum was appropriate” (4.4), and it provided “excellent preparation for a career in simulation-based medical education” (4.5). The fellows rated the faculty highly and felt they were “knowledgeable in the subject matter” (4.7), and effective in “clarity of communication” (4.5), “challenging my thinking” (4.4), and “stimulating enthusiasm” (4.6).

FEAST sessions that received the highest scores were on: debriefing skills/techniques (4.6 out of 5; 1=poor, 5=excellent), debriefing the debriefer (4.5), crisis resource management skills (4.4), interdisciplinary team simulation training (4.2), and research in simulation (4.00). The sessions that received the lowest scores were: audiovisual equipment (3.4), conflict resolution (3.4), and history of simulation in medical education (3.2).

Pre-post Self Assessment Questionnaires

Retrospective pre-post self-assessment questionnaires showed that knowledge and skills in all categories improved. The areas that showed the greatest gains were in meta-debriefing (pooled mean change +2.6), debriefing (+2.5), and simulation centre operations (+2.5). The areas of least improvement were in conflict resolution (+1.4), knowledge of surgical skills props and moulage (+1.5), and knowledge of human factors (+1.6).

Focus Groups and Free-text Survey Comments

Themes arising from focus groups and free text comments on the feedback survey demonstrated that participants appreciated faculty expertise and the structure, practicality, and content of the curriculum. Suggestions for curriculum improvement centered on moving certain topics earlier in the year and proposed lecture topics for subsequent years (Table 4).

**Table 4:** Free-text Comments from Focus Groups and Feedback Surveys

Free-text Comments from Focus Groups and Feedback Surveys
Faculty expertise “Instructors were experts in their topics and had experience to share.” “Discussions generated and moderated by multidisciplinary (health care) professionals.” “Subject matter experts from multidisciplinary backgrounds … presented a rich portrayal of simulation education.”
Structure, Practicality and Content of the Curriculum “Every session was relevant to what I do as a simulation educator – there was very little “fluff” – excellent.” “Interactive sessions and small group discussions.” “Relevant topics linked to practice.” “Learning how to debrief and having this lesson repeated throughout the course.” “The principles of adult learning – understanding that this is what we will be doing and the instructors taking the time to share their knowledge and experiences.”
Suggestions for Improvement “Discussion of debriefing techniques earlier in the year.” “How to manage a difficult debrief.” “Research session as soon as possible.” “More topics about simulation based research.” “More on interprofessional education – how to build scenarios.” “Sessions on using other sim technologies within your scenario design (i.e. hybrid sim, standardized patients).” “I would love to see a formal journal club be a part of FEAST.”

## Discussion

Simulation-based education has gained popularity, yet it must be guided by sound educational theory and practice. The longitudinal nature of the FEAST curriculum allows fellows to gain a profound understanding of topics in educational learning theory underlying simulation-based education, research and techniques for developing and delivering simulation curricula. The success of the program can be attributed to dedicated faculty educators, explicit avenues of communication and strong collaboration between clinical departments and medical education units. FEAST graduates are equipped with the knowledge and skills to develop and conduct simulation-based educational interventions. Some graduates have become consultant staff at our institution; as new FEAST faculty, they give back to the curriculum and perpetuate its evolution. Other benefits of our longitudinal model include sustained networking and “cross-pollination” between fellows and faculty at our institution. This collaboration has facilitated the development of additional interdisciplinary and interprofessional simulation-based sessions. Table 5 lists key practice points for educators wishing to implement a similar curriculum at their institution.

**Table 5:** Key Practice Points for FEAST Curriculum Development and Implementation

Key Practice Points for FEAST Curriculum Development and Implementation
A formal, longitudinal curriculum covering the fundamentals of simulation-based education provides a practical and theoretical foundation for the aspiring simulation educator.
Success requires collaborative leadership to engage multiple disciplines, administrative support and a critical mass of engaged, motivated, and experienced faculty.
Partnership with existing Medical Education Units ensures that educational activities are delivered according to adult-learning theory.
Ongoing benefits of this curriculum include the stimulation of interdisciplinary and interprofessional collaboration.
Graduates have become consultant staff at our institution and have joined the FEAST faculty thereby perpetuating its growth.

An important aspect of any educational intervention is evaluation of its effectiveness. Evaluation of this curriculum has been directed at level 1 of Kirkpatrick’s model, satisfaction with and perception of the curriculum [7]. In the future, moving to higher levels with pre- and post-written and performance-based assessments (level 2, learning) and portfolios (level 3, behaviour) would further bolster credibility of the program.

## Conclusions

We present the successful implementation of a longitudinal simulation educator curriculum that adheres to a well-described framework of curriculum development. The program evaluation shows that FEAST has increased participant knowledge in key areas relevant to simulation-based education and that the curriculum has been successful in meeting the needs of novice simulation educators. Future directions will involve demonstrating impact of the curriculum at higher levels of the Kirkpatrick’s model.

## Appendices

**Table 6:** FEAST Curriculum Themes & Session Learning Objectives*

FEAST Curriculum Themes & Session Learning Objectives
Orientation
Welcome to your Fellowship in Simulation & Medical Education To explain the structure of the DIME Fellowship in Simulation/Medical Education To review the expectations of the DIME Fellowship in Simulation/Medical Education
Introduction to Audio Visual & Patient Simulation Equipment To identify the capabilities and limitations of mannequin platforms To acquire a baseline knowledge of uOSSC capture and playback software/hardware To acquire a baseline knowledge of uOSSC audiovisual/information technology systems and demonstrate how to operate them
Introduction to Simulation Centre Administration & Operations To explain the importance of a clearly defined simulation centre organizational structure To explain the roles and responsibilities of the uOSSC administrative staff and personnel To define the uOSSC funding structure
Educational Principles & Learning Theory
History of Simulation in Medical Education To identify the key driving forces in medical simulation To describe how simulation has evolved over the last 40 years To name the key players and centres in medical simulation
Educational Theory and Simulation To describe five components of “education” To describe the role and importance of “learning to teach” in academic centres To apply the four components of Kolb’s cycle of learning to simulation-based education To describe the strengths and weaknesses of simulation as an educational tool
Curriculum Design in Simulation To develop behaviour-based learning objectives according to best practices To compare and contrast entrustable professional activities and milestones for medical education To select instructional activities and assessment methods to complement learning objectives To describe three barriers to curriculum implementation
Designing Needs Assessments To explain the key elements of a needs assessment, including the importance of triangulation, gap diagnosis, and its potential solutions To list program objectives based on concepts of cognitive psychology, clinical medicine, educational modalities, and assessment instruments
Conducting Programmatic Evaluation To list the sequence of activities within your program To explain the steps in building a limited logic model
High Stakes Assessment in Simulation To explain the factors involved in simulation for high stakes assessment To describe the application of simulation for high stakes assessment
Simulation Instructor Training
Principles of Crisis Resource Management (CRM) To define the principles of CRM To appraise the evidence supporting CRM training To describe techniques for assessment of CRM performance
Simulation Scenario Development To identify learning objectives that address specific training needs best taught using simulation To design a simulation scenario that addresses specific learning objectives To describe the importance of a structured approach to curriculum design and curriculum document generation
Debriefing I: How to Survive Your First Debrief To identify the purpose and goals of the debrief To explain the instructor’s role in the debrief To explain the PEARLs debriefing framework To apply the PEARLs debriefing framework in simulated debriefs with your peers
Debriefing II: Theory in Practice To describe three techniques that can be used during the analysis phase of a debrief To define “debriefing with good judgment” To demonstrate the “advocacy/inquiry” method of focused facilitation To practice debriefing using pre-recorded videos
The Difficult Debrief To identify cues during a simulation scenario and the debrief that forecast a difficult debrief To categorize difficult debriefs according to learner behaviour and debriefer reaction To apply corrective strategies to rescue a difficult debriefing
Debriefing the Debriefer To explain different techniques for assessment of debriefing To gain experience in providing constructive criticism to peer debriefers To rate as valuable the feedback received from others and the process of providing feedback to others
Half-Marathon Debrief the Debriefer (a practical session delivered at the midpoint of the fellowship year) To practice the skills of debriefing the debriefer To analyze debriefing sessions with the purpose of identifying elements that led to a successful debrief To analyze debriefing sessions with the purpose of identifying elements that could be improved upon in challenging debriefs
Marathon Debrief the Debriefer (a practical session delivered at the end of the fellowship year) To practice the skills of debriefing the debriefer To analyze debriefing sessions with the purpose of identifying elements that led to a successful debrief To analyze debriefing sessions with the purpose of identifying elements that could be improved upon in challenging debriefs
Simulation-based Team Training
Interprofessional Simulation To list three factors necessary for developing and implementing an interprofessional simulation session To list three potential challenges unique to interprofessional simulation To design an interprofessional simulation scenario
Interdisciplinary Simulation To design simulation scenarios that simultaneously address the learning objectives of multiple medical specialties (including competencies of collaborator, health advocate and leader) To implement interdisciplinary simulation scenarios that involve participants from multiple different specialties To acquire skills in pre-briefing and debriefing interdisciplinary simulation scenarios involving participants from multiple different specialties
Conflict Resolution Techniques To define conflict To identify sources of conflict To explain techniques for conflict resolution using examples from healthcare environments
Human Factors & Human Error To define two different approaches to error: personal and systemic To list four characteristics of highly reliable organizations and how they may apply toward error management To demonstrate the ability to analyze cases of error using the systemic approach
Technical Skills Simulation
Introduction to Surgical Skills Models, Props & Moulage To explain the steps to build, set-up and run a skills-based simulation session To identify props used in a skills-based simulation session To create some basic models used for a skills-based simulation session
Introduction to Technical Skills Simulation To name factors involved in the choice of a simulation model To explain components of deliberate practice To name different tools used to assess technical skills
Research Skills
Research in Medical Education I (delivered at the beginning of the fellowship year) To explain the principles of medical education research To practice formulation of a research question To provide opportunities to discuss topics for individual research projects
Research in Medical Education II (delivered two months into the fellowship year) To explain key points of research methodology To identify resources to assist in conducting a research project To provide opportunities to discuss the progress of individual research projects
Research in Medical Education III (delivered in the second half of the fellowship year) To provide opportunities to discuss the progress of individual research projects To explain tips and tricks for research dissemination (manuscript writing, submission, and the peer review process)
